# Impact of myocardial iron loading on right ventricular function

**DOI:** 10.1186/1532-429X-11-S1-P162

**Published:** 2009-01-28

**Authors:** Francisco Alpendurada, Monica Deac, John-Paul Carpenter, Paul Kirk, Dudley Pennell

**Affiliations:** Royal Brompton Hospital, London, UK

**Keywords:** Leave Ventricular Ejection Fraction, Right Ventricular, Cardiovascular Magnetic Resonance, Thalassemia, Deferoxamine

## Introduction

Iron-overload cardiomyopathy is a potential complication of transfusion-dependent chronic anemic patients, resulting in significant morbidity and mortality. Beta-thalassaemia major is the commonest cause of iron-overload cardiomyopathy worldwide, representing a major health problem in endemic areas, where heart failure due to iron overload is the principal cause of death.

Cardiovascular magnetic resonance (CMR) has emerged as a non-invasive technique to quantify the amount of iron in the heart. T2-star (T2*) levels correlate with left ventricular (LV) ejection fraction and can predict patients who will develop heart failure. However, correlation between myocardial T2* and right ventricular (RV) function has not yet been established.

## Purpose

To evaluate the relationship of myocardial T2* and RV ejection fraction (RVEF), as well as the relationship between RVEF and occurrence of heart failure within one year, in a population of patients with thalassemia major.

## Methods

We studied 326 consecutive patients (average age: 26.5 years, 45% males) with beta-thalassemia major who were referred for their first scan for assessment of iron loading status by CMR. All patients were on Desferal^®^ (deferoxamine) therapy only at presentation. Those taking other forms of chelation therapy were excluded. RV volumes and RV ejection fraction (RVEF) were calculated and compared with myocardial T2* measured within the interventricular septum. Other variables included LV volumes, left ventricular ejection fraction (LVEF), and the occurrence of heart failure within 1 year of the scan.

## Results

**Figure 1:**
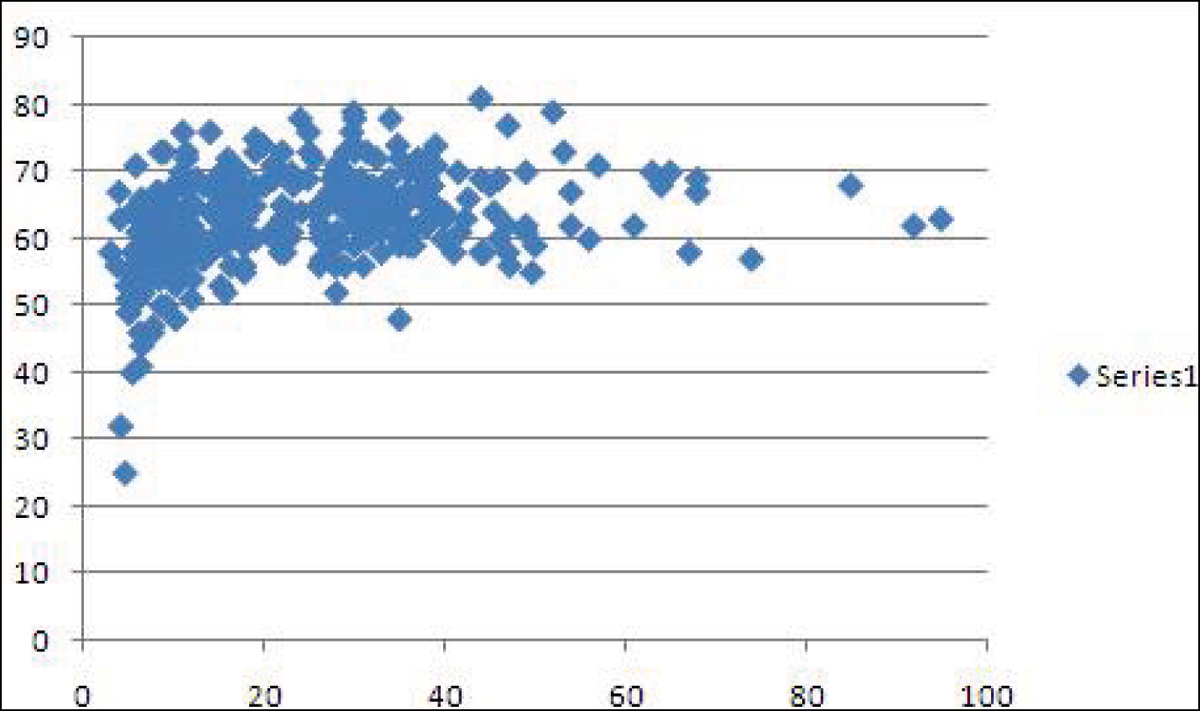
Figure 1

There was a curvilinear relationship between heart T2* levels and RVEF (Figure 1). As T2* decreased to critical levels (indicating significant myocardial iron loading), there was a decline in RV ejection fraction (r = 0.43, p < 0.001). There was also a significant difference (p = 0.0091) in RVEF between patients who developed heart failure within one year of the scan (RVEF: 55.8 ± 11.8%), compared to patients who did not subsequently suffer from episodes of heart failure (RVEF: 63.2 ± 6.8%). Furthermore, we noted a direct linear relationship between the LVEF and the RVEF (r = 0.69, p < 0.001) in these patients.

## Conclusion

Myocardial iron deposition as assessed by CMR is associated with RV dysfunction, which is related with the development of heart failure. This mirrors the decrease in LV function seen with worsening cardiac iron loading, which has previously been described.

Therefore, iron-overload cardiomyopathy not only affects the LV, but also involves the RV to a similar extent. These parameters can subsequently be used as a guide to the management of this condition.

